# *MDM2* SNP309 rs2279744 Polymorphism and Gastric Cancer Risk: A Meta-Analysis

**DOI:** 10.1371/journal.pone.0056918

**Published:** 2013-02-25

**Authors:** Yong Ma, Jianmin Bian, Hongyong Cao

**Affiliations:** Department of General Surgery, Nanjing First Hospital, Nanjing Medical University, Nanjing, People's Republic of China; Sapporo Medical University, Japan

## Abstract

**Background:**

MDM2 is a major negative regulator of p53, and a single nucleotide polymorphism (SNP) in the *MDM2* promoter region SNP309 has been demonstrated to be associated with an increased *MDM2* expression and a significantly earlier age of onset of several tumors, including gastric cancer. Several studies were published to evaluate the association between SNP309 and gastric cancer risk. However, the results remain conflicting rather than conclusive.

**Objective:**

The aim of this study was to assess the association between the *MDM2* SNP309 polymorphism and gastric risk.

**Methods:**

We performed a meta-analysis to investigate this relationship. Odds ratios (ORs) and 95% confidence intervals (CIs) were used to assess the strength of the association. The pooled ORs were performed for codominant model, dominant model, and recessive model, respectively.

**Results:**

Five published case-control studies, including 1,621 gastric cancer cases and 2,639 controls were identified. We found that the *MDM2* SNP309 polymorphism was associated with a significantly increased risk of gastric cancer risk when all studies were pooled into the meta-analysis (GG versus TT, OR = 1.54; 95%CI = 1.04–2.29, and GG versus GT/TT, OR = 1.49, 95%CI = 1.30–1.72). Furthermore, Egger's test did not show any evidence of publication bias (*P* = 0.799 for GG versus TT).

**Conclusion:**

Our results suggest that the *MDM2* SNP309 polymorphism may be a low-penetrant risk factor for the development of gastric cancer.

## Introduction

Gastric cancer is the fourth most commonly diagnosed cancer and the second leading cause of cancer death worldwide [1]. As other complex diseases, gastric cancer is a complex trait caused by both genetic and environmental factors [2]. A large number of epidemiological studies have confirmed the effects of low consumption of fresh fruits and vegetables, high consumption of salty food, smoking, and *Helicobacter pylori* (*H. pylori*) infection on the development of gastric cancer [3]. In addition to above exogenous factors, genetic polymorphisms in the carcinogen detoxification, antioxidant protection, DNA repair and cell proliferation processes are also crucial in the development of gastric cancer [4], [5]. The tumor suppressor p53 is a principal mediator of many cellular functions, including cell cycle, apoptosis, inhibition of angiogenesis and cellular senescence [6]. Somatic mutations that inactivate the p53 gene have been found in at least half of all human solid tumors, highlighting a crucial role of the p53 protein in carcinogenesis [7]. MDM2 is a crucial negative regulator of p53 through several mechanisms. MDM2 directly binds to p53, resulting in the inhibition of p53 transactivation activity [8]. MDM2 also acts as an ubiquitin protein ligase and controls p53 by targeting it for proteasomal degradation [9].

A single nucleotide polymorphism in the *MDM2* promoter, SNP309 T>G (rs2279744), was identified [10], and shown to result in overexpression of *MDM2* RNA and protein. It was further demonstrated that the heightened level of *MDM2* was mediated by and dependent on transcription factor Sp1. Interestingly, amplification of *MDM2* alone can enhance tumorigenesis [11], raising the possibility that the G allele of SNP309 may represent a cancer predisposing allele in its own right.

Recently, a number of studies have reported the role of *MDM2* SNP309 polymorphism in gastric cancer risk, but the results are inconclusive, partially because of the possible small effect of the polymorphism on gastric cancer and the relatively small size in each of the published studies. Therefore, we carried out a meta-analysis on all eligible case-control studies to estimate the overall gastric cancer risk of *MDM2* SNP309 polymorphism as well as to quantify the between-study heterogeneity and potential bias.

## Materials and Methods

### Identification and eligibility of relevant studies

We searched the electronic literature PubMed and Embase for all relevant articles (the last search update was Aug 10, 2012, using the search terms “*MDM*2,” “polymorphism,” and “gastric cancer”). The search was limited to English-language papers. Additional studies were identified by a hand search of the references of original studies. Of the studies with the same or overlapping data published by the same investigators, we selected the most recent ones with the largest number of subjects. Studies included in our meta-analysis have to meet the following criteria: (a) evaluation of *MDM2* SNP309 polymorphism and gastric cancer risk, (b) use a case-control design, and (c) have available genotype frequencies for both patients and control populations.

### Data extraction

Two investigators independently extracted the data and reached consensus on all items. For each study, the following data were considered: author name, year of publication, country of the study, ethnicity, and numbers of genotyped cases and controls. Different ethnic descents were categorized as European and Asian.

### Statistical Analysis

Crude odds ratios (ORs) with 95% confidence intervals (CIs) were used to assess the strength of association between the *MDM2* SNP309 polymorphism and gastric cancer risk. The pooled ORs were performed for codominant model (GG versus TT, GT versus TT), dominant model (GG/GT versus TT), and recessive model (GG vs. GT/TT), respectively. In consideration of the possibility of heterogeneity across the studies, a statistical test for heterogeneity was performed based on the Q statistic [12]. If the *P*>0.05 of the Q-test which indicates a lack of heterogeneity among studies, the summary OR estimate of each study was calculated by the fixed-effects model (the Mantel-Haenszel method). Otherwise, the random-effects model (the DerSimonian and Laird method) was used. Sensitivity analyses were performed to assess the stability of the results, namely, a single study in the meta-analysis was deleted each time to reflect the influence of the individual data set to the pooled OR. Funnel plots and Egger's linear regression test were used to provide diagnosis of the potential publication bias. All analyses were done with Stata software (version 8.2; StataCorp LP, College Station, TX), using two-sided *P* values.

## Results

### Characteristics of Studies

**Figure 1:**
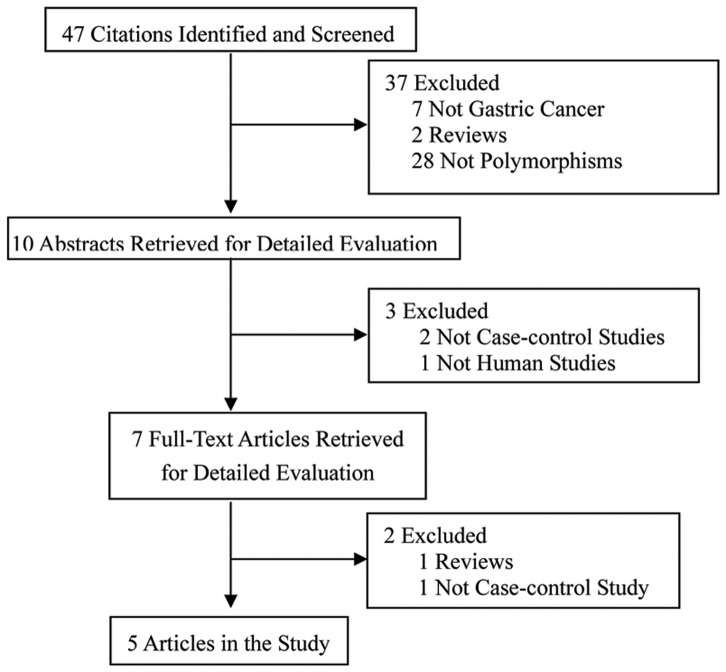
Studies identification, inclusion and exclusion.

There were 47 published papers relevant to the search terms (**Fig. 1**). Through the step of screening the title, 37 of these literatures were excluded (7 were not on gastric cancer, 2 were reviews, and 28 were not polymorphisms). Abstracts from 10 papers were reviewed and an additional three studies were excluded, leaving seven studies for full publication review. Of these, two articles were excluded (one was review, and one was not case-control study). A total of five eligible studies involving 1,621 cases and 2,639 controls were included in the pooled analyses [13], [14], [15], [16], [17]. The characteristics of eligible studies are summarized in **Table 1**. These five studies were all Asian descendants. Gastric cancers were confirmed histologically or pathologically in most studies. A classic polymerase chain reaction-restriction fragment length polymorphism assay was performed in all studies. The distribution of genotypes in the controls of each study was in agreement with Hardy-Weinberg equilibrium except for one study [13].

**Table 1:** Characteristics of literatures included in the meta-analysis.

First author	Year	Country	Ethnicity	Sample size (cases/controls)	HWE^a^	Cases			Controls		
						TT	GT	GG	TT	GT	GG
Ohmiya	2006	Japan	Asian	410/438	0.036	98	188	124	99	241	98
Yang	2007	China	Asian	500/1000	0.877	107	250	143	298	498	204
Cho	2008	Korea	Asian	239/299	0.680	64	110	65	61	152	86
Wang	2009	China	Asian	260/260	0.057	74	120	66	82	141	37
Cao	2007	China	Asian	212/642	0.300	21	91	100	117	299	226

a HWE, Hardy-Weinberg equilibrium.

### Main results

**Figure 2:**
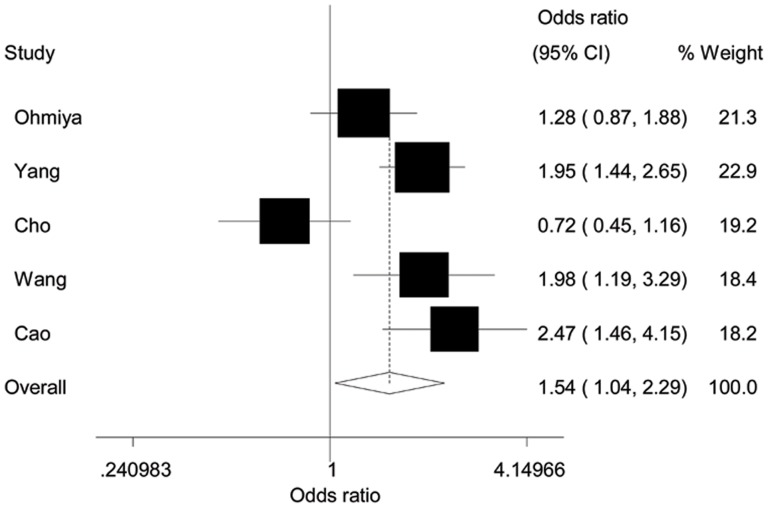
Forest plot of gastric cancer risk associated with the *MDM2* SNP309 (GG vs. TT). The squares and horizontal lines correspond to the study-specific OR and 95% CI. The area of the squares reflects the study-specific weight (inverse of the variance). The diamond represents the summary OR and 95% CI.

As shown in **Table 2**, the variant homozygote GG was associated with a significantly increased risk of gastric cancer, compared with wild-type homozygote TT (OR = 1.54; 95%CI = 1.04–2.29, **Fig. 2**). Furthermore, the *MDM2* SNP309 polymorphism was associated with a significantly increased risk of gastric cancer in recessive model (GG versus GT/TT, OR = 1.49, 95%CI = 1.30–1.72), but not in dominant model (GG/GT versus TT, OR = 1.18, 95%CI = 0.84–1.65).

**Table 2:** Meta-analysis of the *MDM2* SNP309 T>G polymorphism on gastric cancer.

Contrast (n = 5)	OR^a^	(95% CI)	*P*	*P* b
GG vs. TT	1.54	1.04–2.29	0.032	0.002
GT vs. TT	1.03	0.75–1.42	0.844	0.006
GG/GT vs. TT (dominant)	1.18	0.84–1.65	0.332	0.001
GG vs. GT/TT (recessive)	1.49	1.30–1.72	<0.001	0.073

a Random-effects model was used when *P* value for heterogeneity test <0.05; otherwise, fix-effects model was used.

b *P* value of Q-test for heterogeneity test.

### Heterogeneity and sensitivity analyses

**Figure 3:**
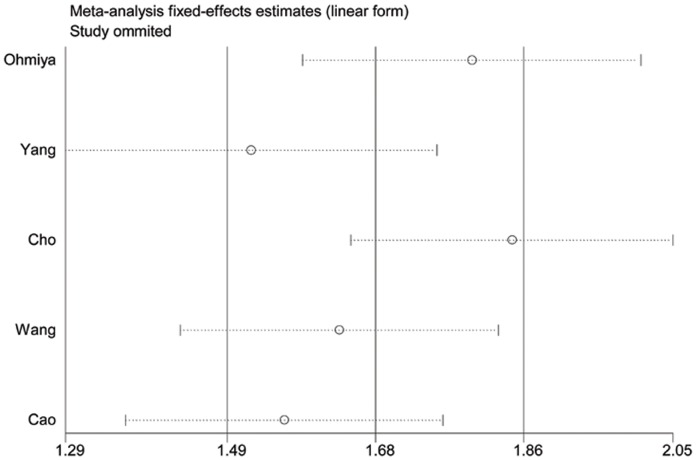
Influence analysis for *MDM2* SNP309 GG vs. TT in the overall meta-analysis. This figure shows the influence of individual studies on the summary OR. The middle vertical axis indicates the overall OR and the two vertical axes indicate the pooled OR when the left study is omitted in this meta-analysis. The two ends of the dotted lines represent the 95% CI.

Significant heterogeneity between studies was observed in overall comparisons except for recessive model (*P*_heterogeneity_ = 0.073 for GG versus GT/TT). Influence analysis was performed to assess the influence of each individual study on the pooled OR by sequential removal of individual studies. The results suggested that no individual study significantly affect the pooled ORs (**Fig. 3**).

### Publication bias

**Figure 4:**
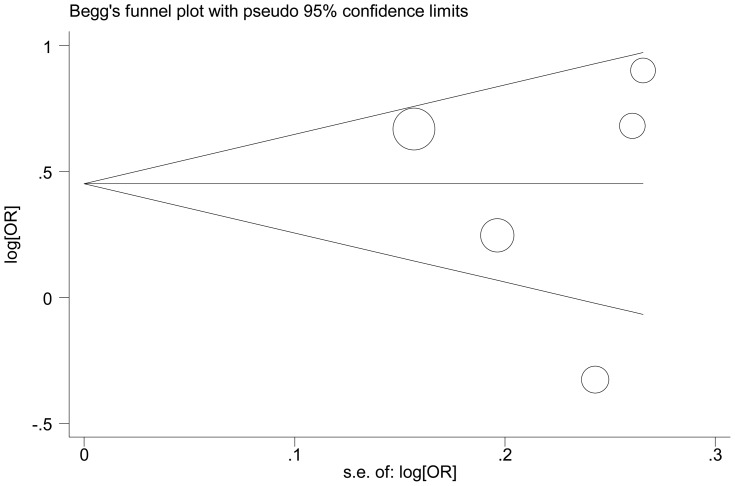
Begg's funnel plot for publication bias test (GG vs. TT). Each point represents a separate study for the indicated association. Log[or], natural logarithm of odds ratio. Horizontal line, mean effect size.

Begg's funnel plot and Egger's test were performed to assess the publication bias of literatures. The shapes of the funnel plots did not reveal any evidence of obvious asymmetry in all comparison models. Then, the Egger's test was used to provide statistical evidence of funnel plot symmetry. The results still did not show any evidence of publication bias (t = −0.28, *P* = 0.799 for GG versus TT, **Fig. 4**).

## Discussion

The previous study has conflicting results about the association between the *MDM*2 SNP309 polymorphism and the risk of gastric cancer, which might be influenced by relatively small sample size and different genetic background. Ohmiya et al. [13] and Yang et al. [14] reported that subjects with variant G allele in the *MDM2* SNP309 polymorphism had an increased risk of gastric cancer. Given the important roles of MDM2 in the regulation of p53, it is biologically plausible that *MDM2* polymorphism may modulate the risk of gastric cancer. The *MDM2* SNP309 polymorphism increases the affinity of Sp1 for the *MDM2* promoter and causes overexpression of *MDM2*. Accordingly, the cell lines with the SNP309 GG and TG genotypes expressed higher levels of *MDM2* than those with the TT genotypes [10]. However, Cho et al. indicated that there was no association between the *MDM2* SNP309 polymorphism and gastric cancer risk [15]. Meta-analysis is a powerful method for resolving inconsistent finding with a relatively large number of subjects. In order to resolve this conflict, a meta-analysis of five studies involving 1,621 cases and 2,639 controls was conducted to derive a more precise estimation of the association. Our results suggest that the *MDM2* SNP309 polymorphism is associated with a significantly increased risk of gastric cancer.

Some limitations of this meta-analysis should be addressed. First, misclassifications on disease status and genotypes may influence the results, because cases in several studies were not confirmed by pathology or other gold standard method, and the quality control of genotyping was also not well-documented in some studies. Second, selection bias could have played a role because the genotype distribution of this polymorphism among control subjects deviated from the Hardy-Weinberg equilibrium in one study [13], and all of the controls were from hospital which may not be representative of the general population. Third, our results were based on unadjusted estimates, while a more precise analysis should be conducted if individual data were available, which would allow for the adjustment by other co-variants, including environmental factors and other lifestyle.

In spite of these, our meta-analysis also had some advantages. First, although the number of studies involved in the meta-analysis was relatively small, the number of total cases and controls were substantial, which significantly increased the statistical power of the analysis. Second, the quality of case-control studies included in current meta-analysis was satisfactory and met our inclusion criterion. Third, we did not detect any publication bias indicating that the whole pooled result should be unbiased.

In summary, this meta-analysis provided evidence of the association between *MDM2* SNP309 polymorphism and gastric cancer risk, supporting the hypothesis that the SNP309 polymorphism may be a low-penetrance susceptibility marker of gastric cancer. However, large sample studies are warranted to validate our findings. Moreover, more gene-gene and gene-environment interactions should also be considered in future analysis, which should lead to better, comprehensive understanding of the association between the *MDM2* SNP309 polymorphism and gastric cancer risk.

## References

[1] Jemal A, Siegel R, Xu J, Ward E (2010) Cancer statistics, 2010. CA Cancer J Clin 60: 277–300. 20610543 10.3322/caac.20073

[2] Crew KD, Neugut AI (2004) Epidemiology of upper gastrointestinal malignancies. Semin Oncol 31: 450–464. 15297938 10.1053/j.seminoncol.2004.04.021

[3] Zhang ZF, Kurtz RC, Yu GP, Sun M, Gargon N, et al (1997) Adenocarcinomas of the esophagus and gastric cardia: the role of diet. Nutr Cancer 27: 298–309. 9101561 10.1080/01635589709514541

[4] Geng JS, Song HT, Wang WR (2004) [Diversity of invasiveness and matrix metalloproteinases expression profile of human gastric carcinoma xenografted in different tissue environments]. Zhonghua Bing Li Xue Za Zhi 33: 53–56. 14989930

[5] Gonzalez CA, Sala N, Capella G (2002) Genetic susceptibility and gastric cancer risk. Int J Cancer 100: 249–260. 12115538 10.1002/ijc.10466

[6] Moll UM, Petrenko O (2003) The MDM2-p53 interaction. Mol Cancer Res 1: 1001–1008. 14707283

[7] Olivier M, Hussain SP, Caron de Fromentel C, Hainaut P, Harris CC (2004) TP53 mutation spectra and load: a tool for generating hypotheses on the etiology of cancer. IARC Sci Publ 247–270. 15055300

[8] Haupt Y, Maya R, Kazaz A, Oren M (1997) Mdm2 promotes the rapid degradation of p53. Nature 387: 296–299. 9153395 10.1038/387296a0

[9] Bond GL, Hu W, Levine A (2005) A single nucleotide polymorphism in the MDM2 gene: from a molecular and cellular explanation to clinical effect. Cancer Res 65: 5481–5484. 15994915 10.1158/0008-5472.CAN-05-0825

[10] Bond GL, Hu W, Bond EE, Robins H, Lutzker SG, et al (2004) A single nucleotide polymorphism in the MDM2 promoter attenuates the p53 tumor suppressor pathway and accelerates tumor formation in humans. Cell 119: 591–602. 15550242 10.1016/j.cell.2004.11.022

[11] Lundgren K, Montes de Oca Luna R, McNeill YB, Emerick EP, Spencer B, et al (1997) Targeted expression of MDM2 uncouples S phase from mitosis and inhibits mammary gland development independent of p53. Genes Dev 11: 714–725. 9087426 10.1101/gad.11.6.714

[12] Handoll HH (2006) Systematic reviews on rehabilitation interventions. Arch Phys Med Rehabil 87: 875. 16731227 10.1016/j.apmr.2006.04.006

[13] Ohmiya N, Taguchi A, Mabuchi N, Itoh A, Hirooka Y, et al (2006) MDM2 promoter polymorphism is associated with both an increased susceptibility to gastric carcinoma and poor prognosis. J Clin Oncol 24: 4434–4440. 16983111 10.1200/JCO.2005.04.1459

[14] Yang M, Guo Y, Zhang X, Miao X, Tan W, et al (2007) Interaction of P53 Arg72Pro and MDM2 T309G polymorphisms and their associations with risk of gastric cardia cancer. Carcinogenesis 28: 1996–2001. 17638920 10.1093/carcin/bgm168

[15] Cho YG, Choi BJ, Song JH, Kim CJ, Cao Z, et al (2008) No association of MDM2 T309G polymorphism with susceptibility to Korean gastric cancer patients. Neoplasma 55: 256–260. 18348658

[16] Wang X, Yang J, Ho B, Yang Y, Huang Z, et al (2009) Interaction of Helicobacter pylori with genetic variants in the MDM2 promoter, is associated with gastric cancer susceptibility in Chinese patients. Helicobacter 14: 114–119. 19751436 10.1111/j.1523-5378.2009.00712.x

[17] Cao Y, Zhang X, Guo W, Wang R, Ge H, et al (2007) Association of theMDM2 polymorphisms with susceptibility of esophageal squamous cell carcinoma and that of ga stric cardiac adenocarcinoma. Tumor 27: 628–632.

